# Oh, the Places You Will Go? Exploring the Geographic Program Distribution and Use of Geographic Preferences in the Radiation Oncology Residency Application Cycle

**DOI:** 10.1016/j.adro.2025.101746

**Published:** 2025-02-22

**Authors:** Mary T. Mahoney, Laura E. Flores, Anthony Alanis, Joshua Y. Qian, Drew T. Bergman, Jie Jane Chen, Stephanie E. Weiss, Jillian R. Gunther, Jeremy G. Price

**Affiliations:** a Department of Radiation Oncology, Fox Chase Cancer Center, Philadelphia, Pennsylvania; b Department of Internal Medicine, University of Nebraska Medical Center, Omaha, Nebraska; c School of Medicine, University of Texas Rio Grande Valley, Edinburg, Texas; d Transitional Year Residency, Memorial Sloan Kettering Cancer Center, New York, New York; e Transitional Year Residency, Newton-Wellesley Hospital, Newtown, Massachusetts; f Department of Radiation Oncology, University of California, San Francisco, California; g Department of Radiation Oncology, University of Texas MD Anderson Cancer Center, Houston, Texas

There is great interest in improving the Electronic Residency Application Service (ERAS) application process for residency selection in the United States (US). Over the past decade, the average number of applications per applicant has climbed across all participating medical specialties, even doubling in many fields.^1^ This phenomenon is known as “application inflation” and “application fever,” where applicants intentionally submit an increasing number of applications as a presumed strategy to maximize interview offers and matching outcomes.^1^ The COVID-19 pandemic has only encouraged this behavior as virtual interviews removed the cost and time burdens associated with traditional in-person interviews, which served as a downstream protective factor against overapplication.^2^^,^^3^

Application inflation is ultimately unsustainable and detrimental to both applicants and programs. The cost of matching, which encompasses application fees and interview costs, soars with the number of applications.^1^ This is expensive for both parties; one study found the average cost for a matched applicant in ophthalmopathy in 2018 was $6684, and the cost to the interviewing department was $3736 per applicant.^4^ Additional application burden on program directors (PDs) threatens increased reliance on standard screening metrics when delineating which applications to review. For example, one anesthesiology PD expressed that if they spent only 10 minutes per application without breaks during their designed protected time, this would still amount to >10 weeks needed to review the 1000 applications they routinely receive.^5^ Because similar measures (United States Medical Licensing Examination [USMLE] scores, clerkship grades, etc) are used by all programs when screening applications, this contributes to the maldistribution of interview offers to the same pool of applicants. This phenomenon has been documented across multiple specialties regardless of competitiveness, with 7% to 26% of applicants accounting for 50% of interview offers.^6^ Given this phenomenon, models have found that increased applications and interview offers paradoxically increase both the number of unfilled positions and unmatched applicants.^2^

A mechanism of signaling genuine interest during the time of application has been proposed as one potential solution. The geographic preferences feature in ERAS allows applicants to voluntarily signal up to 3 of the 9 US census divisions as preferred regions for residency training.^7^ Only programs that share a geographic region that an applicant signaled will “see” the preference – others will not receive any preference information. For example, if an applicant signals both the Middle Atlantic and South Atlantic divisions, a program located in the Middle Atlantic will receive the Middle Atlantic signal but not the South Atlantic signal. An applicant can select “no preference,” which will be received by all programs. An applicant can opt out of geographic signals by leaving the section blank, and no programs receive any preference information.

Pilots of this feature across specialties have shown increased odds of applicants receiving interviews from programs in their preferred geographies^7^, ^8^, ^9^, ^10^ and influence on decisions of program rank lists.^10^^,^^11^ This feature was new to radiation oncology (RO) for the ERAS 2023-2024 cycle. The use of geographic signaling by RO applicants and the distribution of RO residency programs across the 9 census regions have not been previously described. We explored the manner in which RO applicants used geographic preferences for the first time and aimed to advise programs on the possible implications of these designations during residency application review. In this editorial, we highlight recent ERAS data from the initial uses of signaling in RO, discuss the intended implications of signaling, and provide guidance to programs about the best practices for engaging with the signaling tool.

## Geographic Distribution of RO Residency Program and Positions

The 2023 National Resident Matching Program (NRMP) Main Residency Match report lists the location and number of positions offered by all RO residency programs accredited by the Accreditation Council for Graduate Medical Education.^11^ Program locations were categorized using the 9 US census divisions.^7^^,^^12^ Population data for each region were derived from the US Census Bureau database.^12^

**Figure 1:**
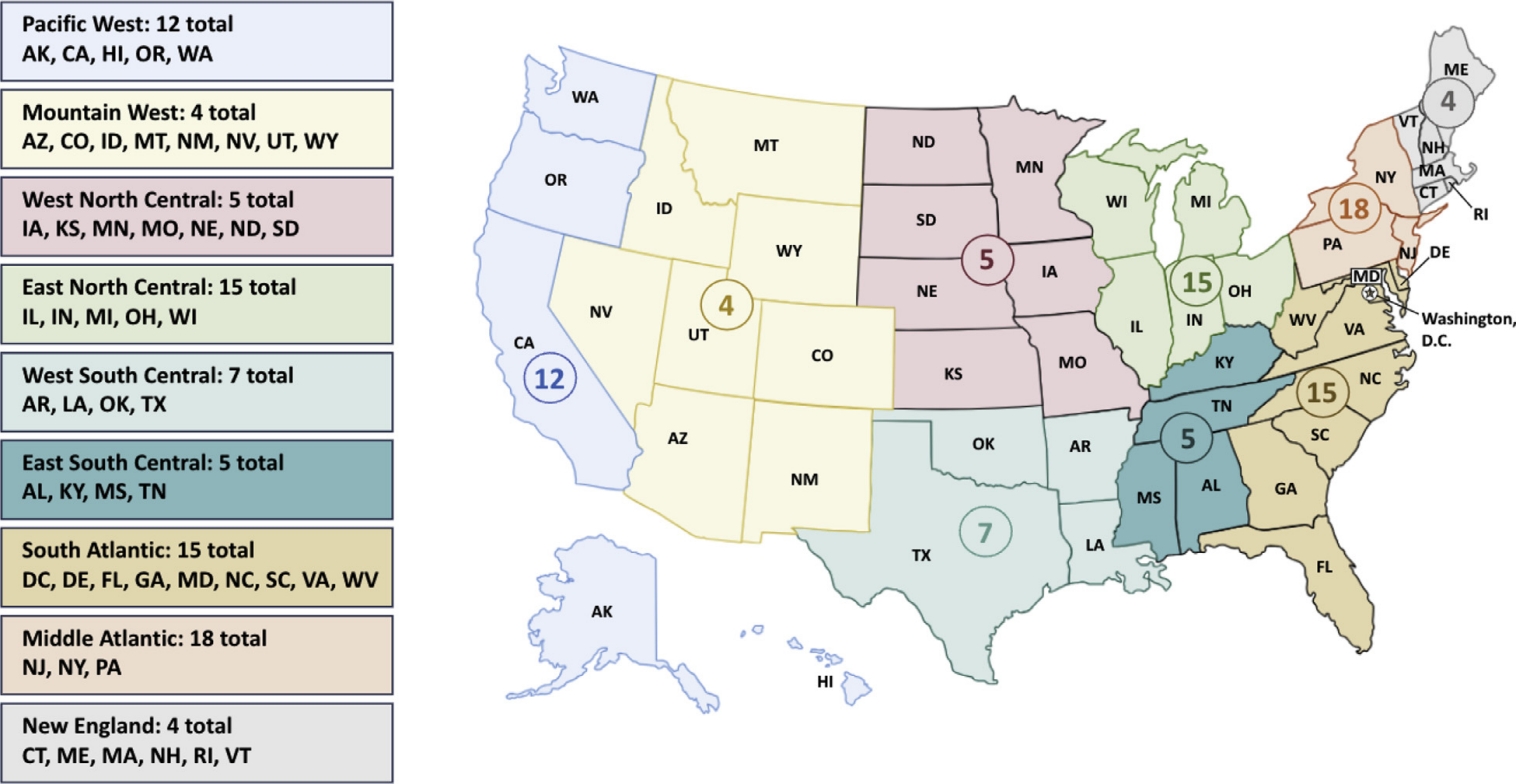
Map of radiation oncology residency programs by US Census Geographic Division in the 2023 Match. *Abbreviations:* AK = Alaska; AL = Alabama; AR = Arkansas; AS = American Samoa; AZ = Arizona; CA = California; CO = Colorado; CT = Connecticut; DC = District of Columbia; DE = Delaware; FL = Florida; GA = Georgia; GU = Guam; HI = Hawaii; IA = Iowa; ID = Idaho; IL = Illinois; IN = Indiana; KS = Kansas; KY = Kentucky; LA = Louisiana; MA = Massachusetts; MD = Maryland; ME = Maine; MI = Michigan; MN = Minnesota; MO = Missouri; MS = Mississippi; MP = Northern Mariana Islands; MT = Montana; NC = North Carolina; ND = North Dakota; NE = Nebraska; NH = New Hampshire; NJ = New Jersey; NM = New Mexico; NV = Nevada; NY = New York; OH = Ohio; OK = Oklahoma; OR = Oregon; PA = Pennsylvania; PR = Puerto Rico; RI = Rhode Island; SC = South Carolina; SD = South Dakota; TN = Tennessee; TT = Trust Territories; TX = Texas; UT = Utah; VA = Virginia; VI = Virgin Islands; VT = Vermont; WA = Washington; WI = Wisconsin; WV = West Virginia; WY = Wyoming.

There were 85 RO residency programs spanning all 9 census divisions, with 204 spots offered for the 2023 Match (Fig. 1). The concentration of programs found in each census division was as follows: Middle Atlantic (18/85, 21%), East North Central (15/85, 18%), South Atlantic (15/85, 18%), Pacific (12/85, 14%), West South Central (7/85, 8%), West North Central (5/85, 6%), East South Central (5/85, 6%), New England (4/85, 5%), and Mountain West (4/85, 5%). There was a statistically significant difference in the number of programs in the Middle Atlantic (18/85, 21%) compared with the Mountain West or New England regions (4/85, 5%) (*P* < .001).

Table 1 delineates the effect of population density by comparing the percentage of the population in each region^1^ to the number of available RO residency spots. More RO residency positions were located within the Middle Atlantic (41/204, 20%) and South Atlantic (36/204, 19%) compared with Mountain West (6/204, 3%) (*P* < .001).

**Table 1:** Proportion of US population and radiation oncology residency spots by region

US census geographic region	Population per region	% Of the total US population	% Of RO residency spots per region
East North Central: IL, IN, MI, OH, WI	47,146,039	14	18
East South Central: AL, KY, MS, TN	19,700,801	6	4
Middle Atlantic: NJ, NY, PA	41,823,740	12	20
Mountain: AZ, CO, ID, MT, NV, NM, UT, WY.	25,716,830	8	3
New England: CT, ME, MA, NH, RI, VT	15,159,777	4	7
Pacific: AK, CA, HI, OR, WA	53,179,975	16	15
South Atlantic: DE, DC, FL, GA, MD, NC, PR, SC, VA, WV	71,431,574	21	19
West North Central: IA, KS, MN, MO, NE, ND, SD	21,763,244	6	6
West South Central: AR, LA, OK, TX	42,198,606	12	9

*Abbreviations:* AK = Alaska; AL = Alabama; AR = Arkansas; AZ = Arizona; CA = California; CO = Colorado; CT = Connecticut; DC = District of Columbia; DE = Delaware; FL = Florida; GA = Georgia; HI = Hawaii; IA = Iowa; ID = Idaho; IL = Illinois; IN = Indiana; KS = Kansas; KY = Kentucky; LA = Louisiana; MA = Massachusetts; MD = Maryland; ME = Maine; MI = Michigan; MN = Minnesota; MO = Missouri; MS = Mississippi; MT = Montana; NC = North Carolina; ND = North Dakota; NE = Nebraska; NH = New Hampshire; NJ = New Jersey; NM = New Mexico; NV = Nevada; NY = New York; OH = Ohio; OK = Oklahoma; OR = Oregon; PA = Pennsylvania; PR = Puerto Rico; RI = Rhode Island; RO = radiation oncology; SC = South Carolina; SD = South Dakota; TN = Tennessee; TX = Texas; US = United States; UT = Utah; VA = Virginia; VT = Vermont; WA = Washington; WI = Wisconsin; WV = West Virginia; WY = Wyoming.

US population data were gathered from the 2023 Census, available at the US Census Bureau.^12^

## RO Applicant Geographic Signal Patterns

The Association of American Medical Colleges website archives aggregated deidentified applicant data from the ERAS 2023-2024 cycle.^13^ Of all the 2024 RO applicants (n = 273), 68% provided at least 1 geographic signal, and the use of this feature varied by applicant type (*P* < .016). A majority of Doctor of Osteopathic Medicine (DO) applicants used all 3 available geographic preferences (85%, 22/26), while 37% (32/87) of international medical graduates (IMGs) tended to opt out of geographic preferences altogether. United States Allopathic Medical Doctor (USMD) applicants (n = 160) were the only applicant type to opt for 2 (n = 11/160, 7%) or a single (n = 10/160, 6%) geographic preference.

Table 2 highlights the distribution of the geographic preference signaling across the 9 census regions. The regions receiving the most signals were consistent with those with the most RO residency positions.

**Table 2:** Distribution of the radiation oncology residency programs, spots, and 2023-2024 Electronic Residency Application Service radiation oncology applicant geographic preference signaling across the 9 US census regions

US census geographic region	Total ERAS geographic preference signals (N = 510) N, %	% Of RO residency programs (n = 85)	% Of RO residency positions (n = 204)
New England: CT, ME, MA, NH, RI, VT	52, 19	5	7
Middle Atlantic: NJ, NY, PA	109, 40	21	20
East North Central: IL, IN, MI, OH, WI	82, 30	18	18
West North Central: IA, KS, MN, MO, NE, ND, SD	17, 6	6	6
South Atlantic: DE, DC, FL, GA, MD, NC, PR, SC, VA, WV	105, 38	18	19
East South Central: AL, KY, MS, TN	19, 7	6	4
West South Central: AR, LA, OK, TX	51, 19	8	9
Mountain West: AZ, CO, ID, MT, NV, NM, UT, WY	24, 9	5	3
Pacific: AK, CA, HI, OR, WA	51, 19	14	15

*Abbreviations:* AK = Alaska; AL = Alabama; AR = Arkansas; AZ = Arizona; CA = California; CO = Colorado; CT = Connecticut; DC = District of Columbia; DE = Delaware; ERAS = Electronic Residency Applicant Service; FL = Florida; GA = Georgia; HI = Hawaii; IA = Iowa; ID = Idaho; IL = Illinois; IN = Indiana; KS = Kansas; KY = Kentucky; LA = Louisiana; MA = Massachusetts; MD = Maryland; ME = Maine; MI = Michigan; MN = Minnesota; MO = Missouri; MS = Mississippi; MT = Montana; NC = North Carolina; ND = North Dakota; NE = Nebraska; NH = New Hampshire; NJ = New Jersey; NM = New Mexico; NV = Nevada; NY = New York; OH = Ohio; OK = Oklahoma; OR = Oregon; PA = Pennsylvania; PR = Puerto Rico; RI = Rhode Island; RO = radiation oncology; SC = South Carolina; SD = South Dakota; TN = Tennessee; TX = Texas; US = United States; UT = Utah; VA = Virginia; VT = Vermont; WA = Washington; WI = Wisconsin; WV = West Virginia; WY = Wyoming.

## Unbalanced Geographic Distribution of RO Training Positions

Our observation of the unbalanced geographic distribution of RO residency programs and positions across the US is consistent with the literature.^14^ Over 15 years, there was rapid and unequal growth of RO residency programs and positions in the Northeast and South compared with other regions.^14^ We demonstrated that the highest concentration of RO programs and positions were in the Middle Atlantic and South Atlantic. This distribution could disadvantage programs if applicants are leveraging geographic signaling to maximize interviews and matching. Recall that applicants only have up to 3 signals, but there are 9 total regions. Additionally, only programs located within that signaled division will see the preference, and thus, programs outside of the signaled regions will not be aware of an applicant's chosen preferences.^7^^,^^10^ We found that the top 3 regions receiving the most signals from 2023-2024 RO applicants, which included the Middle Atlantic, South Atlantic, and East North Central, were consistent with those with the most RO residency positions, accounting for 56% of all the RO residency programs and positions. Additional information regarding the applicants’ medical schools and permanent addresses could help identify potential compounding factors for this association.

Ideally, geographic preferences would reflect an applicant's genuine preferences, or lack thereof, for residency training. Geographic signals should correspond to the physical location of support systems, hometowns, and experiences of the applicants. However, as residency applicants are facing ever-increasing competitiveness and application inflation,^15^ there is a concern for “gamification,” where applicants use their signals to maximize interview offers and chances of matching.^10^ Geographic preferences can independently affect key outcomes; for example, internal medicine candidates who submitted at least 1 geographic preference were significantly more likely to match a program within a signaled region.^8^ This pressure is likely intensified by the loss of convenient screening factors such as scored step 1 and the shift to a more holistic application review.^15^ The ERAS 2022-2023 PD survey across specialties demonstrated that 86% used these geographic signals as a screening tool, 74% included geographic signals in a composite filter for holistic review, and 58% sent interview invitations to every applicant who signaled their region.^9^ We advise programs to be mindful of the realities that applicants face when filtering by geographic preferences. We likewise caution against the use of geographic signals to inform rank list discussions, which was reported by 68% of PD survey respondents.^9^

## Differential Signaling Behaviors by Applicant Type

The observation that RO geographic preference behavior varies significantly by applicant type raises additional concerns about potential gamification. Across specialties, applicants who submitted 3 or no geographic preferences had higher total interviews than those who submitted 1 or 2 geographic preferences.^8^ This “strategy” was demonstrated within aggregated ERAS data where at least one-third of applicants had no geographic preference in the 4 of the more competitive specialties and 2 of the specialties with a larger proportion of IMG applicants.^7^ IMG and DO applicants have historically been perceived as “less competitive” applicant types across specialties, including RO. A majority of NRMP surveyed RO PDs from Match 2024 reported rarely extending interview offers and/or ranking DO and IMG (both US and non-US citizens) graduates.^16^ More than 90% of IMG RO applicants either signaled 3 regions (47%) or had no preferences (37%), whereas USMD applicants were the only applicants with a single or 2 geographic preferences. It should be noted that geographic signals are usually offered along with program signals with an additive, positive effect on interview and match rates,^8^ but RO applicants were not offered program signals for the 2024 Match. For the 2025 Match, RO applicants were offered 4 program signals with geographic preferences.^17^ It will be interesting to follow how program signals are used and if the additive effect of geographic preferences with program signals^8^ is also demonstrated in RO.

## Geographic Signals as a “Plus” or “Minus” Application Factor

Our analysis suggests that geographic preferences are an imperfect system and are capable of both potentially benefiting and harming RO applicants. Per appropriate use guidelines by ERAS, geographic signals are only to be used during the delineation of interview offers as a “plus” factor and discouraged as a “screening” tool.^18^ A survey of RO PDs by the NRMP found that 100% of respondents used geographic signals as a factor when deciding who to interview.^16^ This translated to RO applicants receiving more interview offers from programs that aligned with their geographic preference (51%) compared with no preference (32%) or misalignment (29%).^19^ It is unclear if these geographic signals were used to include (“plus”) or exclude (“screen”) RO applicants during interview invitations. Ideally, geographic signals would be an inclusionary tool during holistic review when delineating interview offers by aiding programs in identifying applicants with geographic ties and/or applicants that they may have otherwise overlooked. One radiology residency program found that they interviewed more geographically diverse applicants after the introduction of geographic signals because they could explicitly see applicants’ geographic preferences instead of inferring them.^20^ However, it is unknown if RO PDs used geographic signals as an exclusionary screening filter by extending interview offers selectively to applicants whose geographic signals aligned. Even more alarming is that 33% of respondent RO PDs confessed to using these geographic signals as a determining factor when ranking applicants, despite explicit discouragement by ERAS.^18^

The nebulous role of geographic signals as an advantage and liability may encourage applicants to be untruthful. Many applicants perceive negative consequences of interview offers for failure to align geographic signals to the division of the programs.^21^ This may, in part, explain why the top 3 regions receiving the most signals from 2023-2024 RO applicants, which included the Middle Atlantic, South Atlantic, and East North Central, were also those with the most RO residency positions. Also concerning is that underrepresented in medicine applicants across specialties tend to have lower completion rates of geographic preferences than their White-only counterparts.^7^ Ultimately, better consensus and communication on the use of geographic signals as a “plus” factor may help applicants and programs alike achieve fairer recruitment.

## Disclosures

Jillian R. Gunther discloses grant funding from the National Institutes of Health R25 for the Summer Medical Student Research Program and the General National Institutes of Health, National Cancer Institute Cancer Center Support Grant; honoraria from Osler Institute and University of Maryland Board Course; Radiation Oncology Education Collaborative Study Group Board Membership, outside of submitted work. The other authors declare that they have no known competing financial interests or personal relationships that could have appeared to influence the work reported in this paper.
